# Mutation status analysis of 58 patients with advanced ALK fusion gene positive non small cell lung cancer

**DOI:** 10.1186/s12890-023-02618-x

**Published:** 2023-09-01

**Authors:** Yuan Yang, Baohua Lu, Mingming Hu, Qunhui Wang, Mei Jiang, Tongmei Zhang, Zhe Liu

**Affiliations:** 1 Oncology Department of Beijing Chest Hospital, Capital Medical University, 9 Beiguan Street, Tongzhou District, Beijing, PR China; 2 Central Laboratory, Beijing Obstetrics and Gynecology Hospital, Capital Medical University, 17 Qihelou Street, Dongcheng District, Beijing, PR China

**Keywords:** ALK fusion, NSCLC, Mutation status, Survival

## Abstract

**Purpose:**

To analyze the characteristics and prognostic values of Anaplastic Lymphoma Kinase (ALK) fusion gene partner, gene subtype and abundance in tumor tissues of advanced Non Small Cell Lung Cancer (NSCLC) patients with positive ALK fusion gene and to explore the best treatment mode of ALK-Tyrosine Kinase Inhibitors(TKIs).

**Methods:**

Cases of advanced NSCLC patients with ALK positive confirmed by both Next Generation Sequencing (NGS) and immunohistochemistry were retrospectively collected. The relationships of Overall Survival (OS)/Progression Free Survival (PFS) between different mutation subtypes, mutation abundance, clinicopathological features were analyzed. OS/PFS between different treatment mode of ALK inhibitors were compared.

**Results:**

Fifty-eight patients were enrolled. There were diverse fusion partners. Five subtypes of Echinoderm Microtubule-associated protein-Like 4 gene (EML4)-ALK fusion mutation were detected: V1,V2,V3,V5 and V7. The mutation abundance ranged from 0.13 to 27.77%, with a median of 5.34%. The abundance of V2 and V5 was higher than V1 and V3 respectively. There was no difference in OS between the low abundance group(≤ 5.34%) and the high abundance group(>5.34%) (P = 0.434). PFS of second-generation ALK inhibitors as first-line treatment was longer than that of Crizotinib as first-line (P<0.001). Never smokers had longer OS than current smokers(P = 0.001).

**Conclusions:**

There are differences in abundance between different fusion partners and subtypes in advanced NSCLC with positive ALK. OS is not associated with subtypes, mutation abundance and first line treatment option of either generation of ALK inhibitors. Smoking is a poor prognostic factor.

Currently, lung cancer is the leading cause of tumor related death worldwide [1]. Targeted therapy has changed the treatment mode of Non Small Cell Lung Cancer (NSCLC). In 2007, Manabu Soda et al. first identified the fusion gene formed by Echinoderm Microtubule-associated protein-Like 4 gene (EML4) and Anaplastic Lymphoma Kinase (ALK) in NSCLC cells in the short arm of chromosome 2, which is another oncogenic driver gene different from the mutation of Epidermal Growth Factor Receptor (EGFR) [2]. In recent years, with the development, marketing and clinical application of ALK Tyrosine Kinase Inhibitors (TKIs), the ALK pathway has made a major breakthrough in the field of lung cancer treatment.

ALK fusion gene has a variety of different fusion partners and subtypes. But in the real world, the immunohistochemical (IHC) method but not Next Generation Sequencing (NGS) is most commonly used for screening in patients with advanced NSCLC. So the detail of ALK fusion gene mutations, including fusion partner, EML4-ALK fusion gene subtypes, abundance, and associated gene mutation status is still unclear, and the relationships between molecular pathological characteristics of ALK fusion gene mutations and prognosis are also unknown.

On the other hand, whether recommended by guidelines or clinical practice, we have multiple generations of ALK inhibitors to choose, but it is still controversial whether prognosis is related to the choice of first-line treatment [3] [4].

This study collected the cases of advanced NSCLC patients with ALK fusion gene positive that were confirmed by both of the method of NGS and IHC to analyze the characteristics of ALK fusion gene subtypes, abundance et al. We further analyzed the relationships between different molecular types of ALK fusion gene, clinicopathological characteristics and prognosis, hoping to reveal the mutation status of ALK fusion gene positive advanced NSCLC patients. We also compared the prognoses between different first line treatment options of different generations of ALK inhibitors in order to explore the best choice.

## Objects and methods

### Objects

Clinical data of 66 patients with advanced NSCLC harboring ALK fusion gene positive confirmed by IHC who were hospitalized in Beijing Chest Hospital, Capital Medical University from May 2015 to June 2021 and had complete medical record data were retrospectively collected. All of them had performed NGS test and 58 cases showed positive ALK fusion while 8 cases negative. The data of 58 positive cases were analyzed. This study obtained ethics approval of The Ethics Committee of Beijing Chest Hospital, Capital Medical University. There was no informed consent in this study because authors had no access to information that could identify individual participants during or after data collection so the ethics approval confirmed this study is in line with the waiver the requirement for informed consent.

Inclusion criteria:(1) Pathological tissue was obtained by lung puncture biopsy/bronchoscopic biopsy/supraclavicular lymph node biopsy/thoracoscopic lesion resection and confirmed as NSCLC by histopathology;(2) EGFR, ALK, Tumor Protein 53(TP53),Kirsten Ratsarcoma viral oncogene homolog (KRAS) and other 26 genes and their mutation abundance were tested by NGS and confirmed that ALK fusion gene positive. (3) VENTANA IHC method using D5F3 antibody confirmed that ALK fusion gene was positive; (4) according to the Union for International Cancer Control(UICC) lung cancer staging criteria (version 8) in stage IIIc or IV;(5) All patients first-line were treated with ALK-TKI targeted therapy, including first-generation Crizotinib: 250 mg twice a day; Second generation drug Alectinib: 600 mg twice a day; Second generation drug Ceritinib: 450 mg, once a day; (6) All patients had at least one measurable lesion with regular follow-up records, imaging data and efficacy evaluation.

### Genetic analysis by NGS

The amplicon libraries were constructed using an Ion AmpliSeqTM Library Kit 2.0 (Thermo Fisher Scientific) with 10-30ng Deoxyribonucleic Acid(DNA) input as a template as previously reported [5] [6]. The custom panel consisted of 21 cancer-related genes, such as ALK, EGFR, KRAS and TP53. For Ribonucleic Acid(RNA)-based NGS analysis, total of 30-80ng input of RNA was used as template and the custom panel was used which contained probes spanning fusions, involving Mesenchymal Epithelial Transition(MET), ALK, Rearranged during Transfection(RET), proto-oncogene tyrosine-protein kinase ROS1(ROS1), and Neurotrophic Tyrosine Receptor Kinase (NTRK) with other genes were identified. The libraries were prepared using the one-step Polymerase Chain Reaction(PCR) amplification method and were sequenced on an DA8600 NGS system of Novogene Co., Ltd. with barcoding performed using an Ion Xpress Barcode Adapter 1–96 Kit. The concept of mutation abundance is the ratio of the number of mutated reads at a specific site to the total number of reads at that site. Expressed as a percentage, it represents the proportion of clones with mutations in the sample. The abundance of the fusion gene is calculated according to the read counts actually obtained by NGS sequencer, and the calculation formula is as follows: Number of specific fusion reads/Total number of reads by sequencing. A ratio greater than 0.1% is defined as ALK fusion gene positive. In our study, the percentage of tumor in each sample range from 10 to 20%.

### Efficacy evaluation

According to Response Evaluation Criteria In Solid tumors (RECIST, version 1.1) [7], efficacy evaluation was defined Complete Response (CR),Partial Response (PR), Stable Disease (SD) and Progressive Disease (PD).CR + PR was objective response and CR + PR + SD was disease control. Progression Free Survival (PFS) of a medication was defined as the time from the initiation of treatment with this drug alone until disease progression or death from any cause. Overall Survival (OS) was defined as the time between diagnosis of advanced lung cancer and death from any cause. The end date of follow-up was September 30, 2022.

### Methods

Patients’ age, gender, smoking history, pathological type, ALK fusion gene mutation status (fusion partner gene, fusion gene subtype and abundance of fusion mutation, etc.), each line of treatment and PFS of each treatment plan were collected and recorded. The OS of 58 patients were followed up. The abundance of different mutation subtypes of ALK fusion gene was compared, the relationships between OS and different mutation subtypes, mutation abundance, smoking status were analyzed, and the PFS of different ALK inhibitors were compared.

## Statistical methods

Statistical software SPSS26.0(Armonk, NY: IBM Corp) was used for data analysis. The abundance of gene fusion between different gene subtypes was analyzed by nonparametric test. PFS and OS were calculated by Kaplan-Meier method, and differences between groups were analyzed by Log-rank test. P < 0.05 was considered statistically significant.

## Results

### Clinicopathological features

A total of 58 patients were enrolled in this study, ranging in age from 27 to 86 years old, with a median age of 54.74 years old. The specific clinicopathological features are shown in Table 1.

**Table 1:** Clinicopathological characteristics of patients according to the ALK fusion mutation status

ALK fusion mutation status		Number	Proportion
Age	≤ 60 years	34	58.6%
	> 60 years	24	41.4%
Sex	male	27	46.5%
	female	31	53.5%
Histologic subtype	Adenocarcinoma	55	94.9%
	Adeno-squamous cell cacinoma	1	1.7%
	Squamous cell cacinoma	2	3.4%
Smoking history	Current	17	29.3%
	Never	41	70.7%
Tumor stage	IIIC	4	6.9%
	IV	54	93.1%

ALK: Anaplastic Lymphoma Kinase

### Distribution of ALK fusion partners and EML4-ALK fusion subtypes

In this study, 55 patients had EML4-ALK fusion gene mutations, accounting for 94.9%, and 1 patient had both EML4-ALK and CAP-Gly Domain Containing Linker Protein Family Member 4(CLIP4)-ALK fusion forms, accounting for 1.7%. Kinesin Light Chain 1(KLC1)-ALK fusion gene mutation occurred in 2 cases (3.4%).

Five fusion mutation subtypes, V1, V2, V3, V5 and V7, were detected in EML4-ALK fusion, and the occurrence frequency of each subtype was shown in Table 2. One case contained both V1 and V7 fusion subtypes.

**Table 2:** Fusion genotypes to the EML4- ALK fusion mutation status

Fusion subtypes	Number	Proportion
EML4-ALK E13;A20 (V1)	21	36.2%
EML4-ALK E20;A20 (V2)	4	6.9%
EML4-ALK E6a;A20 E6b;A20 (V3)	26	44.8%
EML4-ALK E18;A20(V5)	5	8.6%
EML4-ALK E14;Del12A20 (V7)	1	1.7%

EML4: Echinoderm Microtubule-associated protein-Like 4, ALK: Anaplastic Lymphoma Kinase

### ALK fusion gene mutation abundance and comparison of gene mutation abundance between different subtypes

The mutation abundance of 58 patients ranged from 0.13 to 27.77%, with a median of 5.34%.

The mutation abundance of KLC1-ALK fusion gene was 14.67% and 15.06%, and the EML4-ALK abundance was 0.13~27.77% with a median of 5.34%. Kruskal-Wallis test confirmed that the abundance of KLC1-ALK was higher than that of EML4-ALK, the difference was statistically significant (P = 0.033).

#### Mutation abundance between different subtypes of EML4-ALK fusion gene

Kruskal-Wallis test confirmed that the mutation abundance of V5 subtype was higher than V1 and V3, and that of V2 subtype was also higher than V1 and V3, and the difference was statistically significant. There was no difference in mutation abundance between V5 and V2 subtypes, nor between V1 and V3. See Table 3.

**Table 3:** The relationship between the types of fusion genes and the abundance of mutations

Fusion subtypes	Test Statistic	Standard Error	Ajusted P Value
V3-V1	4.266	4.785	1.000
V3-V5	-26.081	7.964	0.006
V3-V2	28.731	8.759	0.006
V1-V5	-21.814	8.115	0.0043
V1-V2	-24.464	8.897	0.036
V5-V2	2.650	10.940	1.000

### Accompanying mutations of ALK fusion gene

Among the 58 patients, 42 patients had only ALK fusion without other accompanying mutations.16 patients had associated mutations. Associated mutation distribution: TP53 in 15 cases; KRAS in 2 cases; There were 2 cases of Max dimerization protein 4(MAD4), 1 case of Phosphatidylinositol-3-kinase catalytic subunit α(PIK3CA), 1 case of Fibroblast Growth Factor Receptor 1(FGFR1), 1 case of FGFR3, 1 case of Discoidin Domain Receptor 2(DDR2), 1 case of Serine/ Threonine kinase 11(STK11), 1 case of EGFR mutation by blood test using diagnostic method of fluorescence quantitative deoxyribonucleic acid PCR, and 1 case of KRAS and TP53 double mutation.

### Relationships between clinicopathological features, treatment and prognosis

V1 and V3 subtypes were the subtypes with the largest proportion in EML4-ALK fusion, so the relationship between OS of V1 (21 cases) and OS of V3 (26 cases) was analyzed. There was no statistical difference in OS between V1 and V3 subtype(P = 0.694).

#### Relationship between ALK fusion gene mutation abundance and prognosis

Taking the median mutation abundance 5.34% as cutoff value, they were divided into low abundance group (≤ 5.34%,30 cases) and high abundance group (>5.34%, 28 cases), There was no statistical difference in OS between the low abundance group and the high abundance group (P = 0.434).

#### Relationship between smoking and prognosis

**Fig. 1:**
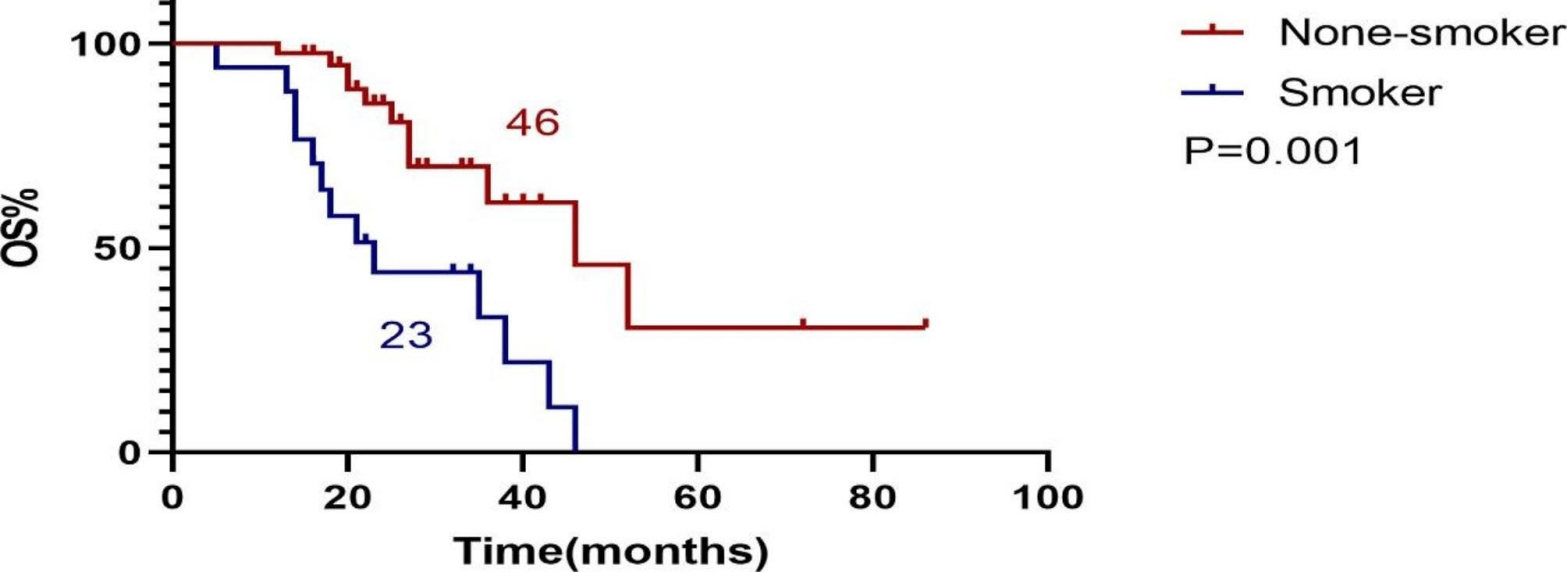
Survival analysis of never smokers VS current smokers. OS: Overall Survival

There were 17 current smokers and 41 never smokers. Survival analysis showed that the OS of never smokers was longer than that of current smokers, and the difference was statistically significant (P = 0.001). See (Fig. 1).

#### Relationships between PFS/OS and treatment mode

A total of 35 patients received first-line Crizotinib therapy, and their PFS was recorded as PFS1.Among them, 27 patients were treated with second-line Alectinib or Ceritinib, after disease progression, and their PFS was recorded as PFS2. Among the 27 patients, 9 patients received rebiopsy after PD and 7 of them had enough tumor tissue to perform NGS again. The result showed 5 cases had ALK fusion still and no resistance mutation was found;1 case had no ALK fusion any more;1 case had ALK fusion still and ALK F1174L point mutation and was treated with second-line Ceritinib.

**Fig. 2:**
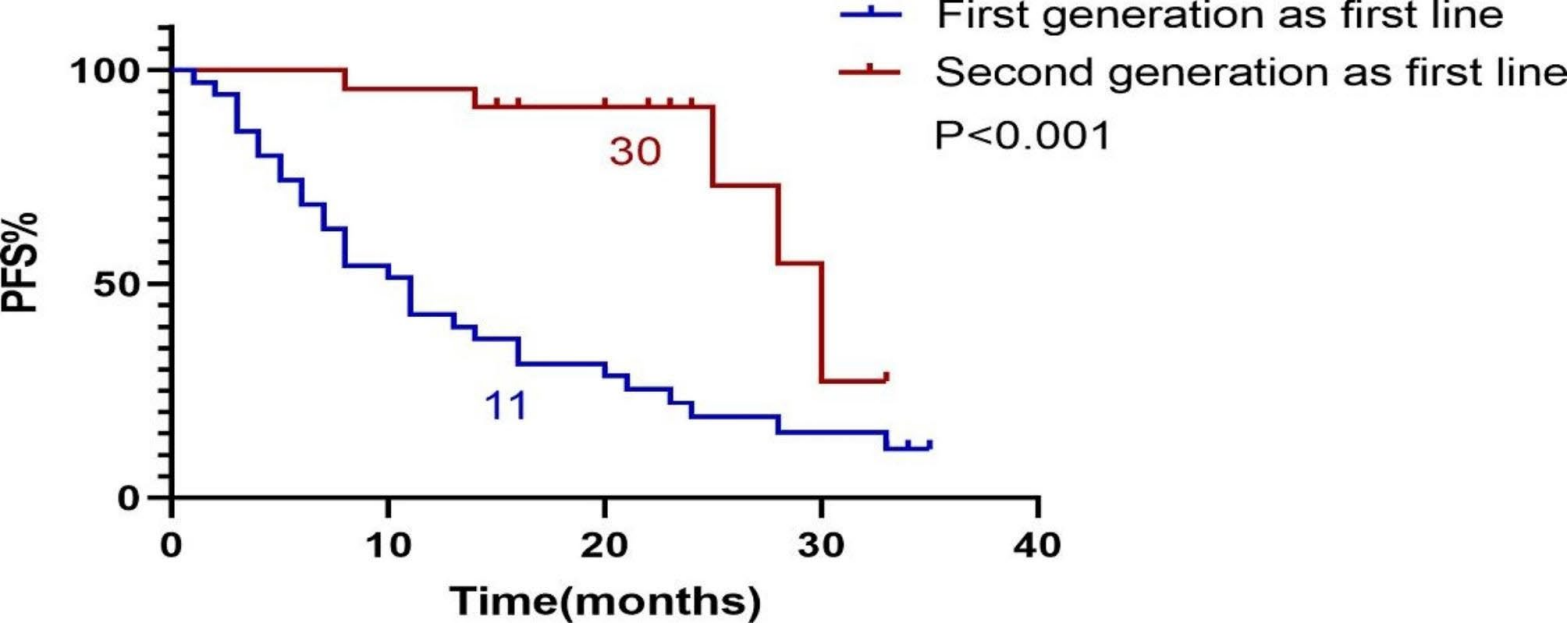
PFS of first-line second-generation ALK inhibitors VS PFS of first-line Crizotinib. PFS: Progression Free Survival. ALK: Anaplastic Lymphoma Kinase

**Fig. 3:**
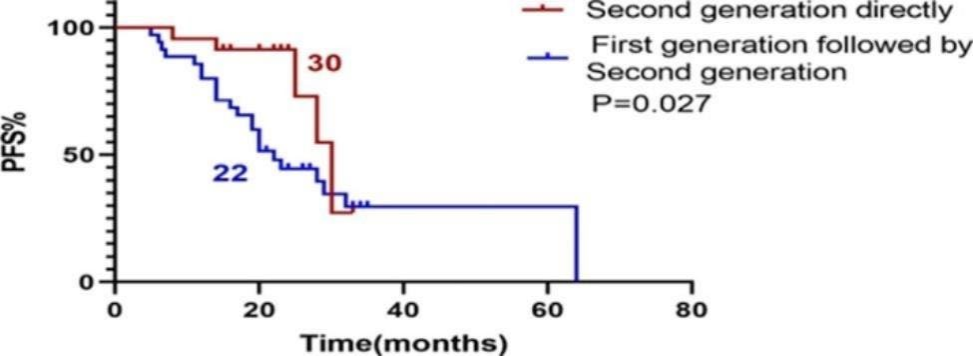
PFS of first-line second-generation ALK inhibitors VS PFS of Crizotinib followed by second-generation ALK inhibitors. PFS: Progression Free Survival. ALK: Anaplastic Lymphoma Kinase

**Fig. 4:**
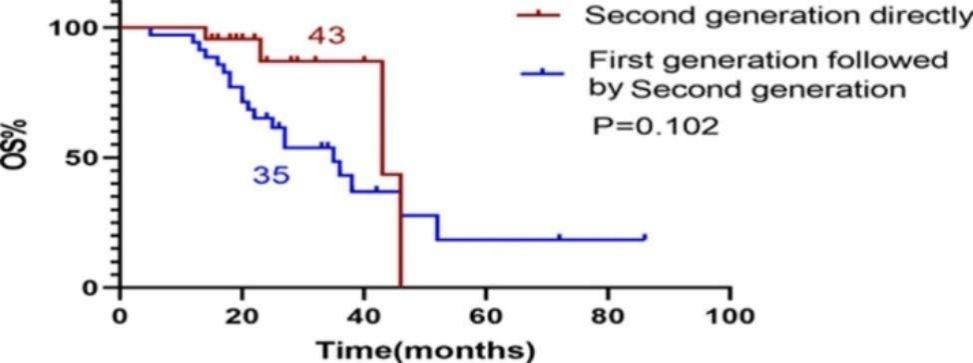
OS of first-line Crizotinib VS first-line second-generation ALK inhibitors. OS: Overall Survival. ALK: Anaplastic Lymphoma Kinase

A total of 23 patients received first-line treatment Alectinib or Ceritinib, and their PFS was recorded as PFSa. We found that PFSa with first-line second-generation ALK inhibitor was longer than PFS1 with first-line Crizotinib (P < 0.001). (Fig. 2). PFSa of first-line second-generation ALK inhibitors was also longer than PFS1 + PFS2 of first-line Crizotinib patients (P = 0.027). (Fig. 3). There was no difference in OS between patients treated with first-line Crizotinib and those treated with first-line second-generation ALK inhibitors (P = 0.102). (Fig. 4).

## Discussion

ALK gene encodes Orphan Receptor Tyrosine Kinase (RTK), which is involved in the differentiation, regeneration and synaptic formation of nerve cells. Normally, ALK gene is only expressed in specific tissues during the embryonic period and promotes cell proliferation and division. It is dormant in adults, does not express proteins and is not carcinogenic [8]. ALK forms a tyrosine kinase active dimer with its fusion partner, Activate ALK and its downstream Rat Sarcoma virus-Mitogen Activated Protein Kinase(RAS-MAPK) and Phosphatidylinositol 3 Kinase/protein kinase B(PI3K-AKT) signaling pathway, thereby causing abnormal cell proliferation and differentiation, forming a powerful tumor driving force, leading to malignant tumor proliferation.

### ALK detection methods

There are a variety of methods to detect the status of ALK. IHC is the prior recommendation to screen by the Molecular Pathology Committee of Chinese Society of Pathology [9]. As IHC is of low price and less time-consuming and it has the highest sensitivity to 94.5–100% [10] [11].It is reported that IHC and NGS have a concordance rate of 84.5–87% [11] [12].In our study, among 66 IHC positive cases, 58(87.9%) were also showed the same result in NGS, which is basically consistent with previous literature.

### Different fusion partners

With the application of NGS, In ALK fusion there are Kinesin Family member 5B(KIF5B)-ALK [13], TRK-fused gene(TFG)-ALK [14], KLC1-ALK [15], etc., more than 90 fusion partners are identified. Although a large number of new fusion partners have been found in ALK fusion gene positive NSCLC, the earliest EML4-ALK fusion form is the most common, accounting for about 85% [16] [17] [18], which is basically consistent with the data in our study. Two cases of KLC1-ALK fusion were found in our study, and the number of previous reports was rare. Wang and his/her colleagues reported a case of lung cancer with both MET gene amplification and KLC1-ALK fusion, this patient had a good response to Crizotinib [19]. Our study also found they both had good response to ALK-TKIs. This shows, KLC1-ALK fusion, a rare fusion type, also is meaningful.

### EML4-ALK fusion gene subtype

To date, more than 17 fusion subtypes with fusion breakpoints in EML4 exons 2, 3, 6, 7, 8, 10, 13, 14, 15, 16, 17, 18, 19, 20, 21 and 23 have been identified in EML4-ALK-positive NSCLC. In addition, small deletions and insertions are found at the EML4-ALK fusion junction [20]. Functionally and clinically, EML4-ALK variants can be divided into “short” types (V3a/b and V5a/b, which do not contain the TAPE domain) and “long” types (which contain part of the TAPE domain), resulting in different degrees of cellular protein stability and different sensitivity to ALK-TKIs [21]. Our study found 5 subtypes in EML4-ALK fusion, which shows the diversity of EML4-ALK fusion.

### Associated gene mutation of ALK fusion gene

Earlier literature reported that ALK fusion gene mutation and EGFR gene mutation were independent molecular events [22] [23] [24].Justin F Gainor et al. retrospectively analyzed the clinical genotyping data of 1687 NSCLC patients and found no cases of coexistence of EGFR mutation and ALK fusion mutation, so it was believed that ALK fusion and EGFR mutation were mutually exclusive [22].However, subsequent literature found that ALK fusion patients could also be combined with EGFR mutations [25] [26], and 5 of 7 patients with double mutations achieved partial response after first-line use of EGFR-TKI [26]. Patients with ALK fusion gene mutation could develop acquired EGFR mutation after Crizotinib resistance [27] [28]. Also, after the application of EGFR-TKI, acquired ALK fusion gene mutation can also occur, leading to drug resistance of EGFR-TKI [29]. This complex phenomena suggests that the mutation of ALK fusion gene is closely related to the mutation of EGFR sensitive gene. In our research, EGFR sensitive mutation was detected in peripheral blood of a patient with subtype EML4-ALKV1 (EGFR mutation was not detected by NGS method), and disease progression occurred only 1.5 months after first-line oral administration of Afatinib, and 44 months of total OS time was obtained after replacement with second-generation ALK-TKI. The reason why EGFR-TKI is ineffective may be related to the high sensitivity of peripheral blood to detect EGFR mutations.

### Clinical characteristics of patients

Previous retrospective literature indicated that ALK positiveness is more common in young and never smoking or light smoking NSCLC patients [30], and ALK fusion gene mainly occurs in lung adenocarcinoma, which is extremely rare in lung squamous cell carcinoma or other histological types of NSCLC [31] [32].The results of our study are consistent with the above conclusions: the proportion of young, adenocarcinoma and never smoking patients is significantly higher, which reflects the specific population distribution characteristics of ALK fusion gene mutation.

### The abundance of ALK fusion gene

The abundance of fusion gene represents the proportion of mutated clones in this sample. we revealed that the abundance of V2 and V5 subtypes was higher than that of V1 and V3 subtypes respectively, and the difference was statistically significant. This indicates that the abundance between different subtypes may vary. In advanced lung adenocarcinoma patients with EGFR 19Del/21L858R mutations, the EGFR mutation abundance may be associated with the outcome when the patients were treated with EGFR-TKIs [33] [34]. In our study, we did not find the prognostic value of the abundance of ALK fusion gene. As the abundance is also associated with proportion of tumor cells in each sample and there is no standardized method to detect the abundance in this day and age, the value of abundance is still unclear.

### Relationships between clinical characteristics and prognosis

Previous retrospective studies showed that the EML4-ALK-V3 subtype predicted short PFS and was more likely to produce solvent front ALK-G1202R mutation after the first/second generation of ALK-TKI therapy. Horn et al. [35] analyzed that Lorlatinib was a better choice for EML4-ALK-V3 subtype in the CROWN test [20] [36]. According to our analysis, the median OS time of V1 subtype patients was 43 months and the OS of V3 subtype patients was 35 months. Although the OS of V3 was shorter, the difference was not statistically significant. Considering the relatively small number of cases with V1 and V3 subtype, the conclusion is worth further exploration.

Current studies on the OS benefit of Crizotinib sequential second-generation ALK-TKI versus direct second-generation ALK-TKI are controversial. A meta-analysis [37] [38] suggests that second/third generation ALK-TKIs such as Alectinib, Brigatinib, Seritinib, and Loratinib may induce longer OS as first-line treatment compared to Crizotinib, but other studies suggested the OS were similar when Crizotinib was first used as long as second/third generation were used sequentially [3] [4]. In our study, The OS of direct application of the second-generation ALK-TKI was longer than that of the first generation sequential second-generation (43 versus 35 months). But the difference was not statistical significant. The ALK fusion gene mutation population may have a long OS and the enrolling period of this study is long (6 years). As second-generation drugs came on the market late, the patients first-line using second-generation ALK-TKI were enrolled late correspondingly. All these above should be considered. Therefore, this study will continue to extend the follow-up time to observe the comparison of OS benefits. In our research, due to drug accessibility, 8 patients used Loratinib in the posterior line (third lines or more lines) and the median PFS of Loratinib was only 8.5 months, suggesting that it may be more beneficial for patients to survive if the drug with good efficacy is pushed to the first line. In our study, the OS of current smoking patients was significantly shorter than that of never smoking patients, and the difference was also statistically significant.

According to the data in our study, the PFS of Crizotinib sequential second generation ALK-TKI was 22 months and the PFS of direct application of second-generation ALK-TKI was 30 months, the difference was statistically significant. In this study, PFS of patients were shorter than clinical trial data, which may be related to the interference of multiple factors, such as the poor PS score of patients in the real world, multiple basic diseases and differences in patient compliance during treatment.

## Conclusion

With the expansion and popularization of NGS detection methods, we can accurately understand the fusion form, fusion subtype and abundance of ALK fusion mutations in NSCLC. There are significant differences in the abundance between different subtypes of fusion genes and fusion partners. Smoking is a poor prognostic factor for ALK fusion gene mutation. The effect of direct administration of second-generation ALK-TKI may be better than that of first-generation Crizotinib sequential second-generation ALK-TKI. This provides real-world evidence for treatment modalities for ALK fusion gene-positive NSCLC patients.

## Data Availability

The datasets used and analysed during the current study are available from the corresponding author on reasonable request.
